# Practicable strategies parents can apply in their daily routine to successfully implement the 50/50-split-model of paid work, childcare, and housework: a qualitative content analysis

**DOI:** 10.1186/s12889-024-19646-9

**Published:** 2024-08-14

**Authors:** Ronja Schaber, Josefine Simm, Tirza Patella, Susan Garthus-Niegel

**Affiliations:** 1 Institute and Policlinic of Occupational and Social Medicine, Faculty of Medicine Carl Gustav Carus, DREAM Studie, Technische Universität Dresden, Fetscherstraße 74, 01307 Dresden, Germany; 2 Institute for Systems Medicine (ISM), Faculty of Medicine, Medical School Hamburg, Hamburg, Germany; 3 Department of Childhood and Families, Norwegian Institute of Public Health, Oslo, Norway

**Keywords:** 50/50-split-model of paid work, childcare, and housework, Dual-earner parents, Gender role distribution, Gender equality, Success strategies, Human families, Parenting, Public health, Qualitative content analysis, DREAM study

## Abstract

**Background:**

Many young couples are planning to share paid work, childcare, and housework equally between each other. But implementing such a 50/50-split-model is difficult and parents often return to traditional gender role distributions after the birth of a child. This return has potential negative effects on mental health, physical health, and relationship satisfaction. Therefore, this study aims to find practicable strategies on a behavioral-level which new parents can apply in their daily routine to successfully implement the 50/50-split-model if they wish to do so.

**Methods:**

This qualitative study, DREAM_TALK_, is part of the multi-method, prospective Dresden Study on Parenting, Work, and Mental Health (DREAM). For DREAM_TALK_, *N* = 25 parents implementing a 50/50-split-model were selected based on quantitative data regarding time use, which participants had provided in questionnaires. In DREAM_TALK_, problem-centered interviews were conducted with the selected sample at 17 months postpartum. Those were analyzed via qualitative content analysis, which is systematic, rule-guided, and based on the criteria of validity and reliability.

**Results:**

The qualitative content analysis revealed a catalog of 38 practicable strategies to manage daily routine, which can help parents to successfully implement a 50/50-split-model. Individual participants used 23 success strategies on average. Examples include having a regular coordination appointment with the other parent, planning foresightedly, flexibility, reducing cleaning, optimization of routes, or moderate split-shift parenting. Some of these strategies seem opposing, e.g., planning foresightedly, and at the same time, meeting unpredicted changes with flexibility. Those seemingly opposing strategies were well balanced by the participants, which was an additional strategy.

**Conclusions:**

Parents can use the success strategies relatively independently of external circumstances. This behavioral perspective extends prior theories, which have focused on explaining unequal gender role distributions with external circumstances. A behavioral perspective can be a gateway to assist more parents to pioneer in implementing the 50/50-split-model, which might in turn lead to a healthier and more satisfied public population.

**Supplementary Information:**

The online version contains supplementary material available at 10.1186/s12889-024-19646-9.

## Background

This article studies an egalitarian gender role distribution, where parents share the three domains paid work, childcare, and housework equally between each other. This means, one parent spends the same amount as the other on each of these three domains. We call this the *50/50-split-model of paid work*,* childcare*,* and housework*, in short, *50/50-split-model*. Many young adults have egalitarian attitudes and wish to implement a 50/50-split-model in their future [1, 2]. Nonetheless, parents achieving its implementation are a minority [3–7]. The aim of this study is to find practicable strategies on a behavioral-level which new parents can apply in their daily routine to successfully implement the 50/50-split-model if they wish to do so. This is a novelty, as previous research has developed important explanations for the unequal gender role distributions but has lacked in providing parents strategies on how to implement more gender equal role distributions.

The following background section will firstly provide data on the scarcity of the 50/50-split-model, secondly report the positive impacts of its implementation for public health, thirdly go through prior literature and its explanations for the unequal gender role distribution, and fourthly unfold the aim of this article by explaining how these theories are missing behavioral-level strategies which can help parents implement the 50/50-split-model.

### The scarcity of the 50/50-split-model

Not all parents are dual-earner parents and even if they are, most do not share the three domains paid work, childcare, and housework equally. If U.S. dual-earner parents are clustered into different groups of dual-earners, only 9–12% are in groups of equal [4] or nearly equal [3] distributions of paid work, childcare, and housework. Zooming in on groups of dual-earner parents with nearly equal distributions, mothers still take on the larger share of unpaid work across many different high-income countries [3, 5, 6]. While childcare is sometimes shared more equally, distribution of housework remains unequal [6]. In Germany, mothers spend around three times more minutes per day on housework than fathers [7].

This is interesting, seeing that around 40–70% of high-income country populations hold egalitarian attitudes [8, 9]. Vignette studies showed that childless, young adults would prefer to live in egalitarian family arrangements and believe they would be more satisfied in those [1, 2]. Moreover, there is no gender specific preference for paid or unpaid work [10]. A German vignette study presented vignette-scenarios of different family models, also including, e.g., paid work being distributed unequally. Across all scenarios, 40% of participants believed that housework should be distributed equally [11]. All these percentages are a lot higher than the percentages of parents actually implementing egalitarian family models. Thus, there seems to be a difference between theoretical preference for, and practical implementation of egalitarian family models.

The practical implementation seems to become especially difficult with the birth of a child. Longitudinal data show that after becoming parents, the gender gap between paid and unpaid work increases, also if parents had relatively egalitarian role distributions or values pre-birth [12, 13]. Liberal (e.g., U.K.) and conservative (e.g., Germany) countries shift from egalitarian values and practices pre-birth to tensions between egalitarian values and inegalitarian practices post-birth. “Gender equality is, thus, upset by the birth of the first child and then followed by an *adaptation to inequality*” [14 p. 57].

In sum, many young adults have egalitarian attitudes and wish to implement a 50/50-split-model in their future [1, 2]. Nonetheless, parents achieving its implementation are a minority [3–7].

### Advantages of the 50/50-split-model for public health

There are many indications in literature that implementing a 50/50-split-model might be healthy for the public population. For women, a high housework overload and an unmet need for spousal support in childcare and housework was associated with lower mental well-being [15, 16] and lower physical health [17]. Perception of inequality in childcare and housework was associated with psychological distress [18] and lower physical health [17]. However, if hours spent on housework and paid work were more equally distributed between husband and wife, depressive symptoms were lower [19]. Moreover, the odds of working mothers developing postpartum depression were significantly lower than those of nonworking mothers [20, 21]. For men, perception of inequality in childcare and housework was also associated with psychological distress [18], whereas if hours spent on housework and paid work were more equally distributed between husband and wife, depressive symptoms were lower [19]. In sum, this research indicates, that more equal distributions of paid work, childcare, and housework might be healthier for women and men.

In addition to mental and physical health, relationship satisfaction seems to be influenced by the division of childcare and housework. Multiple studies have shown that perceived fairness in the division of childcare [22, 23] and housework [22, 24–26] is related to a higher relationship satisfaction of women [22–26] and men [22] only for housework, [23, 25, 26]. Women’s perception of fairness regarding childcare and housework seems to be influenced by more than the equality of its division, e.g., the reference women compare their division to (see theory of distributive justice, e.g., [27]). However, recent studies have shown an association between the equality of the division of housework and the perception of its fairness [25] as well as a direct relationship between the equality of the division of childcare and relationship satisfaction [23]. Additionally, if a parent expected an equal division pre-child and this expectation was violated, this influenced their level of satisfaction [28]. In sum, for parents who wish to implement a 50/50-split-model, being able to do so will likely result in higher relationship satisfaction.

### Predominant explanations for the unequal gender role distribution

A large body of literature tried to explain the gender gap in paid work, childcare, and housework. Four micro-level theories persisted in literature: time availability, relative resources, gender ideology, and gender display. The impact of these theories on the division of paid work, childcare, and housework seems to be moderated by macro-level (e.g., politics, culture) and individual predictors (e.g., sexuality, ethnicity; for reviews, see [29–31]).

The time availability theory posits that the partner who has more time, i.e., the partner who works less in paid work, will do more childcare and housework. The relative resources theory assumes that housework tasks are undesirable, and each partner tries to bargain her-/himself out of doing them. The partner with more relative resources (e.g., income, education) has more bargaining power and will therefore do less housework. In a modification of this perspective, research has looked at absolute resources. This “autonomy approach” assumes that it will be easier for people to bargain for what they want if they are independent individuals, i.e., have enough absolute money to support themselves, even if it is relatively less than their partners [32]. The gender ideology theory posits that housework is divided according to gender role values. If individuals have more traditional values, tasks will be divided more unequal than if values are more egalitarian (for reviews, see [29, 31]). All three theories have received wide empirical support to explain the unequal division of childcare (e.g., [33]) and housework (e.g., [34, 35]). Nonetheless, they have been criticized for not explaining why women persist to do more childcare and housework, even if they work the same or more hours in paid work than their partners, have similar resources, and egalitarian values [29, 36].

The gender display theory posits that women want to be perceived as womanly and men as manly and therefore act accordingly [29]. Some research differentiates gender display into *doing gender* [37] and *neutralizing gender deviance* [38]. When doing gender, individuals reaffirm their gender through gendered actions, e.g., a woman showing her womanhood by worrying about her child. When neutralizing gender deviance, individuals try to reduce gender deviance, e.g., a husband, who earns less than his wife, does even less housework to protect his manhood. Gender display could explain inconsistencies of prior theories, e.g., why women who earn more than their partners still do more childcare and housework. Indeed, it received empirical support for childcare and housework (e.g., [39, 40]) and advanced the understanding of the labor distribution. However, it has been criticized, among other aspects, for not answering the question on how individuals can “undo gender”, i.e., how to reduce gender differences [41].

Studies about the influence of macro-level predictors on the gender division of labor look at the level of egalitarianism of a country via indices like availability of paternal leave or percentage of parliamentary seats held by women. In more egalitarian countries, the division of labor is more egalitarian (e.g., [42, 43]). Moreover, there are multiple indications that the impact of micro-level theories is moderated by macro-level predictors (e.g., [42, 44, 45]). E.g., it might be easier for mothers to use their bargaining power to convince fathers to share childcare equally in early postpartum months if paternal leave is available in the country, as this simplifies the practical realization of early paternal care.

Additionally, reviewers have called for more attention regarding individual predictors, such as sexuality and ethnicity, which might also moderate the explanatory power of micro-level theories [30, 36].

In sum, previous research has tried to solve the puzzle of the gender gap in paid work, childcare, and housework [29–31]. The developed explanations largely contributed to the understanding of the gender gap, but they have never been able to fully explain it and recently been criticized [29, 36].

### Theoretical framework and aim of study

**Fig. 1:**
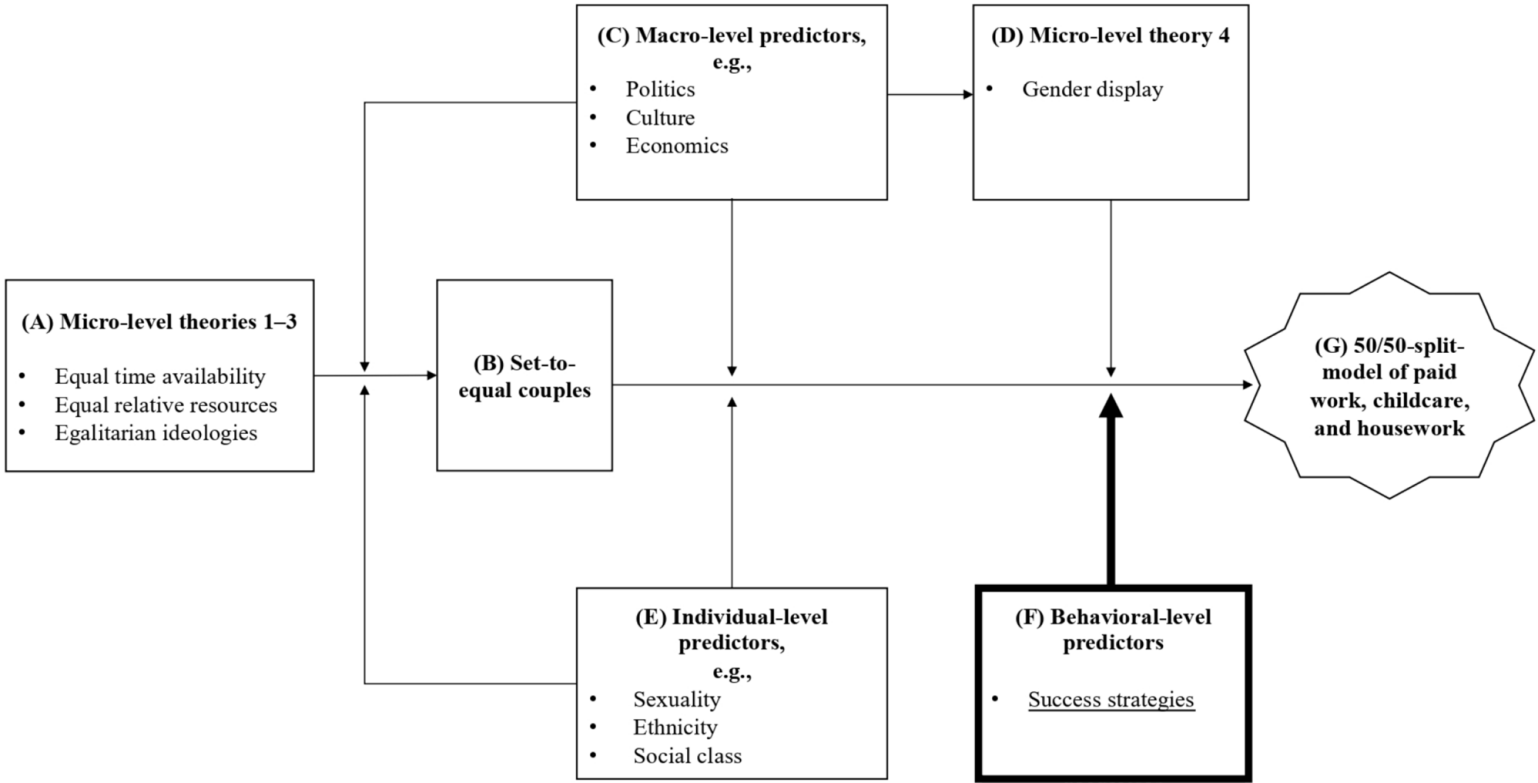
Theoretical framework. Theories and predictors that were developed in prior studies to explain the persisting gender gap in paid work, childcare, and housework (**A**, **C**–**E**) interplay with behavioral-level predictors, i.e., success strategies, developed in the present study (**F**). Together they can explain how a 50/50-split-model of paid work, childcare, and housework, i.e., gender equality at work and at home could be achieved (**G**)

Based on previous research on the division of labor we developed a theoretical framework (Fig. 1), explained in the following. The first three micro-level theories, time availability, relative resources, and gender ideology predominantly explain why couples with pre-birth unequal time availability, resources, and traditional gender ideologies have a traditional division of labor. But many contemporary couples have pre-birth equal time, resources, and egalitarian ideologies (Fig. 1A). We call these *set-to-equal couples* (Fig. 1B). The three theories have been criticized for not explaining why set-to-equal couples do not implement a 50/50-split-model [29, 36].

After being set-to-equal, the second set of predictors, gender display, macro-level, and individual-level predictors might have a more predominant impact (Fig. 1C–E). Via gender display, parents may unconsciously slip into more traditional patterns, even if set-to-equal. Macro-level predictors strongly influence whether a 50/50-split-model is even possible [45]. E.g., if there is no public childcare available (provided by politics), one parent must stay at home and will not be able to participate in paid work. A problem becomes apparent: The second set of predictors does not entail options to actively counter this process (compare [41] regarding gender display). Regarding macro-level predictors like culture, if you are a pregnant, set-to-equal couple, but live in a traditional culture, there is not much you can do about it, except move to a more egalitarian country—an unrealistic and potentially undesirable option. Hence, previous predictors understand individuals who want to implement a 50/50-split-model as passive subjects of their circumstances.

However, individuals’ behavior is not “wholly determined by situational influences. Rather, human functioning is a product of a reciprocal interplay of intrapersonal, behavioral, and environmental determinants” [46 p. 165, 47]. This study aims to find practicable strategies on a behavioral-level which new parents can apply in their daily routine to successfully implement the 50/50-split-model if they wish to do so (Fig. 1F). This way we focus on the ability of the individual rather than the individual as a dependent of environmental determinants. We specifically focus on practicable strategies in the daily routine, relatively independent of external predictors.

Few previous studies had adjacent aims. Müller [48] and Daly [49] looked at time-management strategies of dual-earner parents. Deutsch and Gaunt [50] followed their own call [41] and researched how dual-earner parents sharing childcare and housework equally “undo gender”. Dienhart [51] asked how parents organize their life to involve fathers. Cluley and Hecht [52] focused on work-family decision making of dual-earner parents. However, the inclusion criteria of these previous studies never entailed sharing all three domains—paid work, childcare, and housework—equally. Probably because 50/50-split-model-parents are scarce and hard to find. Moreover, our focus on daily routine is unique. Nonetheless, some similar strategies have previously been reported. Whenever comparable, those will be referred to in the results and discussion section, where we will present our extensive list of success strategies regarding daily routine management.

In sum, the aim of this article is to find strategies on a behavioral-level which new parents can apply in their daily routine to successfully implement the 50/50-split-model. Those need to be practicable and independent of external circumstances to offer a low-threshold gateway for the many young couples who wish to implement the model.

## Methods

### Research design overview

The present qualitative study, DREAM_TALK_, is part of the multi-method, prospective Dresden Study on Parenting, Work, and Mental Health (“**DR**esdner Studie zu **E**lternschaft, **A**rbeit und **M**entaler Gesundheit”; **DREAM**; study protocol, [53]). In the quantitative part of the study, *N* = 3 860 (expectant) parents living in Dresden, Germany complete various questionnaires. The six measurement points are between pregnancy and 4.5 years postpartum (see study protocol for details, [53]). Quantitative data of the third measurement point (14 months postpartum; *SD* = 0.69; range = 13–16) were screened to purposively select participants for the qualitative DREAM_TALK_ interviews.

To address our aim, on the one side, we intend to generate previously unknown and rich data and find potentially latent meaning in this data. On the other side, we intend to reduce this data systematically to create general concepts, which can be easily understood, potentially be applied, and have some degree of transferability. Regarding the philosophical underpinning or research paradigm, we locate these intentions between postpositivism and constructivism (see Ponterotto [54] for guide on locating research within philosophical underpinning). The methodology of DREAM_TALK_ was chosen in accordance with our aim and philosophical underpinning and will be described in the following, starting with participant recruitment, followed by data collection procedures, and finally data-analytic strategies.

### Participant recruitment

Inclusion criteria for DREAM_TALK_ entailed: both partners of a heterosexual couple partaking in the quantitative DREAM study and having filled out the third measurement point (14 months postpartum; *SD* = 0.69; range = 13–16). This timepoint was chosen in line with parental leave rights of Germany. Every mother and father has the right to take up to three years of parental leave, during which her/his job is protected. Parents are supported by the government with parental allowance for up to 14 months [55]. Due to these regulations, at 14 months postpartum, parents decide whether both parents return to work or whether only one parent returns, which, in Germany, is usually the father [56]. Thus, 14 months postpartum is a crossroad, at which parents can set the course for either a 50/50-split-model or a more traditional division of labor. Further inclusion criteria entailed living together permanently as a couple and with the child, and the respective partners spending the same number of hours on paid work, childcare, and housework. For the last criterion, various individually answered questions were merged to create couple data, including time use estimates of hours spent on the three domains (based on [4]) and the subjective feelings of the division of the domains (based on the Akershus Birth Cohort; e.g., [57]). Participants were eligible for contact, if a couple divided each of the domains paid work, childcare, and housework within the range of 40–60% between themselves. By merging couple data, potential biases, including egocentric bias [58] and the finding that men report higher gender equality than women [34] become unproblematic.

After screening, each partner of an eligible couple was approached as an individual, with the possibility of both or only one partaking in DREAM_TALK_. Potential participants were informed via e-mail about the qualitative DREAM_TALK_ interviews and called one week later to confirm whether they were interested in participation and make an interview appointment. Saturation was reached with the collected sample of *N* = 25 [59, 60]. Sample characteristics are shown in Table 1.

**Table 1:** Sample characteristics of participants

Sample characteristics^a, b^	Total (*N* = 25)		
	*n* (%)	M ± SD (Range)	Ratio
Participation of partner^c^			
Partner participates in study	22 (88)		
Partner does not participate in study	3 (12)		
Sex			
Female	13 (52)		
Male	12 (48)		
Country of birth			
Germany	25 (100)		
Other	0 (0)		
Age in years		31.7 ± 2.6 (28–36)	
Education ^d^			
University degree	14 (56)		
No university degree	11 (44)		
Income^e, f, g^			
Own income		1 875 (930–15 000)	
Household income		3 662 (1 875–>20 000)	
Own income : household income			0.51
Household income : average household income of Saxony ^h^			1.77
Marital status			
Married	12 (48)		
Not married	13 (52)		
Number of children			
One child (i.e., toddler ^i^)	22 (88)		
Two children (i.e., toddler^i^ and sibling^j^)	3 (12)		
Toddlers age in months ^i^		17 ± 1.44 (15–20)	
Siblings age in years ^j^		6 ± 1.41 (5–8)	
Expecting another child^k^			
Expecting	5 (20)		
Not expecting	20 (80)		

*Note *^a^ Characteristics at time of interview, if not indicated otherwise. ^b^ Characteristics described for each participant individually, not as a couple. ^c^ Both respective partners of a couple could participate in the study; participants were interviewed and data analyzed individually. ^d^ Measured at T1 (during pregnancy) of the quantitative DREAM study. ^e^ Missing data of one participant. ^f^ Income statistics calculated using median. ^g^ Monthly net income in Euro, including all assets. ^h^ The average monthly net income of households in Saxony in 2019 was 2 068€ [61]. ^i^ Toddler refers to the child that participants were expecting at T1 of the quantitative DREAM study (index child). ^j^ Sibling refers to older children that were born prior to the participation in the quantitative DREAM study. ^k^ In case of male participant, *n* indicates that partner/wife was expecting at time of interview

### Data collection procedures

Problem-centered interviews were chosen as an interview technique, as they are eligible for research topics where some prior knowledge already exists, while at the same time allowing participants to speak about previously unknown factors [62]. This is in accordance with our research topic, as some prior knowledge is available, but we also intend to find previously unknown success strategies. Moreover, this qualitative approach will allow us to generate a rich set of data.

The interview guide was prepared according to Witzel and Reiter [62]. It entailed, e.g., the following opening question on daily routine, aiming to generate a narration: “I would like to understand how you are able to implement the 50/50-split-model. Which factors or people help and where do you maybe experience difficulties? To get a first insight into how your daily life looks, please tell me as detailed as possible about a typical day from morning to evening [translated]”. An example follow-up question was: “How do you plan and organize your daily routine [translated]?”. As problem-centered interviews are semi-structured, the interview guide was only a memory aid. The interviewer mainly followed the topics addressed by the participants [62].

In understanding that qualitative research cannot be completely neutral [63, 64], the researchers aimed to consciously acknowledge their values and understand their roles as observers [65]. Reflexivity was a constant during the whole research project. Thus, the interviewer wrote a postscript after each conducted interview entailing reflexive information, e.g., first impressions, emotions, and potential improvements for the next interview.

The interviews took place from November 2019 to March 2020. They were conducted 17 months postpartum (*SD* = 1.44; range = 15–20). To attain best possible response rates, participants could choose the interview location. Most chose the University. The duration of one interview was, on average, 1:55 h (*SD* = 0:15; range = 1:23–2:26). Each interview took place with one individual participant. Interviews were audio recorded, transcribed verbatim in German, pseudonymized, and inserted into the qualitative data analysis software NVivo [66]. The sample references (R) presented in this article were translated after the analysis.

### Data-analytic strategies

Interview transcripts were analyzed via qualitative content analysis (QCA) following Schreier [67]. QCA is a qualitative method which historically developed out of quantitative content analysis and thus also entails some quantitative elements [67]. On the one hand, the method allows the analysis of large amounts of qualitative data for latent meaning. On the other hand, it entails a systematic reduction of this data and the subsumption of specific information under more general concepts, which can be easily understood and potentially be applied. This composition of qualitative and quantitative elements is an optimal choice for our aim to generate a rich set of previously unknown, practicable strategies on a behavioral-level which new parents can apply in their daily routine to successfully implement the 50/50-split-model.

QCA follows three systematic steps. In the first step, the coding frame was developed. It consists of main-, sub, and sub-subcategories structuring the interview material. Main categories specify relevant aspects and subcategories specify relevant meanings concerning these aspects. Main categories are thus on a higher hierarchical level than subcategories [67]. The categories were developed inductively via progressive summarization and subsumption as described by Schreier [67] and Mayring [68]. For reflexivity, the research team met regularly to discuss the coding frame. Each category of the coding frame was given a name and definition. Thus, in the following steps, text segments of the interview material could be assigned to the different categories, i.e., “coding”. In the second step of the QCA, the coding frame was trial coded via intercoder-coding, to assess the internal reliability of the coding frame. Two researchers independently coded the text segments to the categories of the coding frame and Cohen’s kappa coefficients were calculated as described by Kuckartz [69], to test consistency in coding. All Cohen’s kappa coefficients were equal or over 0.8 (see Table 2). This means, the two independent researchers mostly coded the same text segments to the same categories and the coding frame had a good internal reliability. In the third step of the QCA, during main coding, all relevant interview text segments were assigned to the categories of this coding frame. As a result, we can show how many participants spoke about each sub- or sub-subcategory and how many text segments in sum regarded each sub- or sub-subcategory.

To assess the validity of inductive coding frames, face validity is recommended [67]. Coding frames have a higher face validity if the coding frequency for residual categories (*Others*) is low, the distribution of coding frequencies within one main category is equally distributed, and the coding frame is differentiated. Considering these criteria, the final coding frame has a sufficient face validity, indicating that the categories of the coding frame adequately represent the concepts under study. In sum, the three steps of this QCA reduced data, were systematic, rule-guided, and based on the quality criteria of validity and reliability [67, 70]. This distinguishes QCA from other qualitative methods but makes it very suitable to address our research aim.

In a subsequent step of the analysis, some categories were combined in NVivo (“combination-categories”). This means that NVivo filters for references assigned to a specific combination of subcategories. Combining categories builds on the QCA-rule that one individual text reference may be assigned to multiple subcategories (of different main categories; [67]). For example, if the subcategory *childcare* (and not *household*) is combined with the subcategory *together*, references where *childcare* tasks were done *together* are filtered out and references where *household* tasks were done *together* are omitted. Thus, in the results and discussion section, we can show how many childcare tasks were done together in comparison to how many housework tasks were done together (and not just how many tasks were done together).

## Results and discussion

The final coding frame is presented in Table 2. It shows all main-, sub-, sub-subcategories, including combination-categories, and Cohen’s kappa coefficients. It cross-references 3 080 text segments. It shows how many participants spoke about each subcategory (participant) and how many text segments in sum regarded each subcategory (reference). The number of times a specific subcategory was mentioned allows for an indication on the importance of this subcategory to succeed in implementing the 50/50-split-model. However, even though the QCA following Schreier [67] enabled us to quantify our data, our original data are still qualitative. We thus caution our readers that all following discussions related to the number of participants or references can only be interpreted as indicative.

**Table 2:** Main-, sub-, and sub-subca﻿tegories of the coding frame

Main categories ^a^	CK	Subcategories ^a^ Sub-subcategories	Participants (*N* = 25)		References (*N* = 3 080) ^b^
			*n*	% ^c^	*n*
**1. Foundations for a 50/50-split-model**					
1.1 Mutual agreement to implement the 50/50-split-model	0.84	1.1.1 Mutual agreement	24	96	78
1.2 Desire to implement the 50/50-split-model was a criterion in partner selection	0.84	1.2.1 Relevant criterion	10	40	13
		1.2.2 Ambivalent	1	4	1
		1.2.3 No relevant criterion	3	12	3
		1.2.4 Not mentioned	11	44	0
**2. Daily routine management**					
2.1 Methods for superordinate coordination of tasks and appointments	1	2.1.1 Fixed routines	24	96	145
		2.1.2 Mutual coordination appointment ^d^	9	36	15
		2.1.3 Daily communication	23	92	116
		2.1.4 Coordination specialist	7	28	19
2.2 Strategies applied during coordination of tasks and appointments	0.88	2.2.1 Foresighted planning	23	92	144
		2.2.2 Conscientious use of calendar ^e^	12	48	28
		2.2.3 Flexibility	25	100	183
		2.2.4 Assessing the importance of paid work meetings together ^f^	16	64	38
2.3 Jointly used tools to coordinate tasks and appointments	1	2.3.1 Analog family calendar	17	68	33
		2.3.2 Digital family calendar	9	36	18
		2.3.3 Mobile communication	12	48	21
		2.3.4 Cleaning schedule (written down)	6	24	33
		2.3.5 Others	3	12	7
2.4 Reducing household workload	1	2.4.1 Reducing cleaning	25	100	66
		2.4.2 Reducing cooking	7	28	10
		2.4.3 Reducing grocery shopping trips	7	28	10
		2.4.4 Supporting household appliances	16	64	21
		2.4.5 Others	6	24	6
2.5 Distribution of (remaining) tasks	0.82	2.5.1 Designated responsibility	24	96	197
		2.5.2 Scheduled exchange (timely)	18	72	74
		2.5.3 Situational exchange (spontaneously)	21	84	95
		2.5.4 Together (not distributed)	25	100	97
2.6 Execution of (remaining) tasks	0.95	2.6.1 Optimization of routes	20	80	64
		2.6.2 Rule of “30-seconds”	10	40	19
		2.6.3 Utilizing mini-timeslots	9	36	12
		2.6.4 Working in parallel in the household	13	52	29
		2.6.5 Moderate split-shift parenting	17	68	32
		2.6.6 Combining tasks with leisure	5	20	5
		2.6.7 Others	5	20	9
2.7 Child’s location [combination-category] ^g^	1	2.7.1 Child sleeping ^h^	—	—	—
		2.7.2 Childcare by other parent ^h^	—	—	—
		2.7.3 Childcare by social network ^h^	—	—	—
		2.7.4 Child at daycare ^h^	—	—	—
		2.7.5 Child involved in household chores ^h^	—	—	—
2.8 Assessment of the partner’s childcare and housework abilities	0.91	2.8.1 Positive assessment ^i^	25	100	126
		2.8.2 Component: positive assessment referring to childcare directly postpartum ^i^	13	52	16
		2.8.3 Ambivalent assessment	12	48	20
		2.8.4 Negative assessment	10	40	27
2.9 Regulating emerging imbalances in household and childcare	1	2.9.1 Regulating to prevent own overload	19	76	81
		2.9.2 Regulating to prevent partner overload	17	68	40
		2.9.3 Acceptance of temporary fluctuations	8	32	16
2.10 Task [combination-category] ^g^	1	2.10.1 Childcare ^j^	—	—	—
		2.10.1.1 Childcare: universally	—	—	—
		2.10.1.2 Childcare: bringing/picking-up child to/from daycare	—	—	—
		2.10.1.3 Childcare: in case of sickness	—	—	—
		2.10.2 Household ^j^	—	—	—

*Note* CK = Cohen’s kappa, resulting from trial coding

^a^ All categories created inductively. ^b^ Sum of all references for categories presented in this article, incl. combination-category references. ^c^ Percentage scores exceed 100% because different references from the same participant can be coded into different subcategories. ^d^ Meets the coding criteria of *fixed routine* but only coded as *mutual coordination appointment* to follow the criterion of mutual exclusiveness [67]. ^e^ Meets the coding criteria of *foresighted planning* but only coded as *conscientious use of calendar* to follow the criterion of mutual exclusiveness [67]. ^f^ Meets the coding criteria of *flexibility* but only coded as *assessing the importance of paid work meetings together* to follow the criterion of mutual exclusiveness [67]. ^g^ Combination-categories can only be evaluated in combination with other categories, refer to Tables 3, 4 and 5. ^h^ The criterion of mutual exclusiveness [67] was omitted due to participants sometimes stating more than one potential location of child in the same sentence such as, “child is with my parents or my partner while I do XYZ”; in such cases double coding within the main category was allowed. ^i^ The criterion of mutual exclusiveness [67] was omitted due to overlaps of content by definition of these subcategories; double coding within the main category was allowed. ^j^ The criterion of mutual exclusiveness [67] was omitted due to participants sometimes speaking about their “tasks” universally; in such cases double coding within the main category was allowed

This results and discussion section follows the structure of Table 2 and is grouped into two major parts: (1) Foundations for a 50/50-split-model and (2) daily routine management. Both parts contain subheadings. Each subheading equates to one main category, under which its related *subcategories* (in italic font) and corresponding sample references (R) are discussed. The first part, foundations for a 50/50-split-model, contains important preconditions, before parents can start to implement the 50/50-split-model. In the second part, daily routine management, each *subcategory* represents one success strategy which parents can apply in their daily routine to be able to implement a 50/50-split-model (Fig. 1F).

### Foundations for a 50/50-split-model

**Fig. 2:**
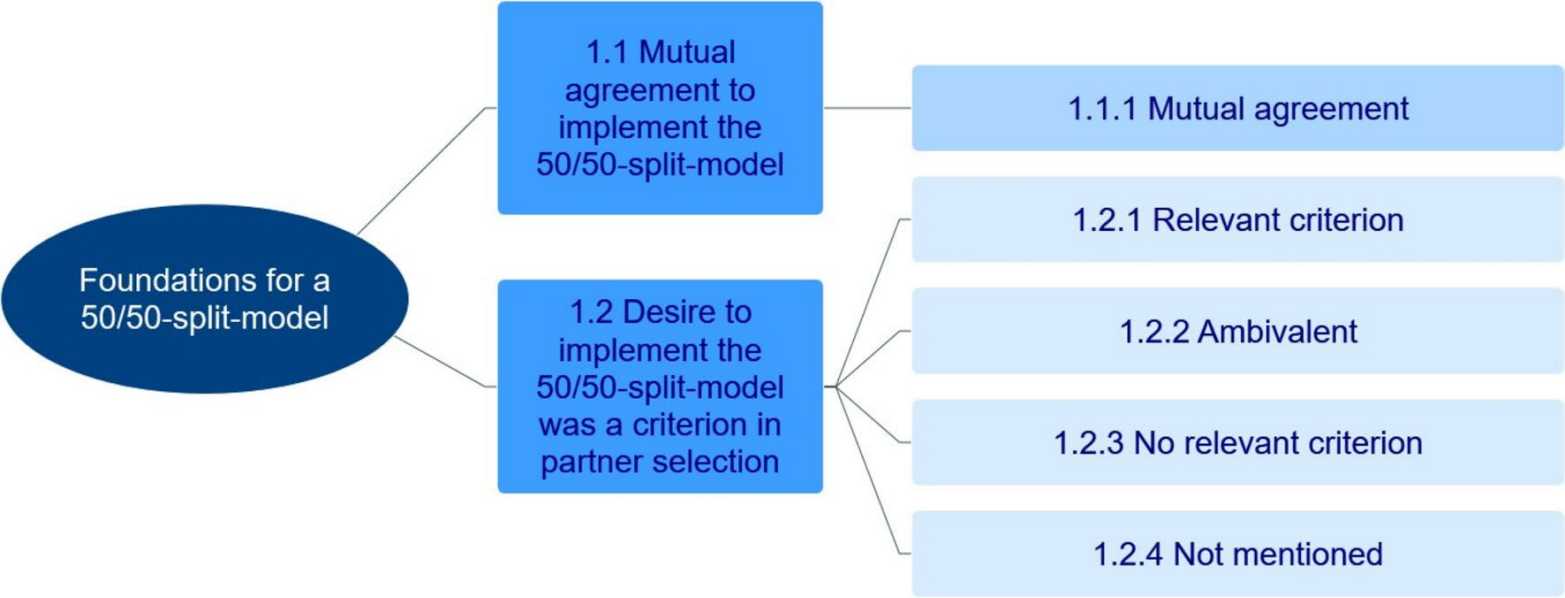
Foundations for a 50/50-split-model. Hierarchical level of main- and subcategories presented from left (higher level) to right (lower level)

The analysis revealed foundations which are necessary before parents can start to implement a 50/50-split-model. For an overview, the hierarchical level of the main- and subcategories regarding these foundations is presented in Fig. 2.

#### Mutual agreement to implement the 50/50-split-model

Before couples can start implementing the 50/50-split-model, they must agree that they want to do so. This agreement can but does not have to be verbally formulated. Important is the fact, that both partners have the desire to implement the 50/50-split-model. Almost all participants spoke about a *mutual agreement to implement the 50/50-split-model* (R1). This is in line with the gender ideology theory, stating that if individuals have egalitarian attitudes, they are more likely to adapt egalitarian role distributions [29]. In our theoretical framework (Fig. 1), we place mutual agreement with set-to-equal, as a foundation before applying daily routine management strategies.

1. Subcategory 1.1.1 Mutual agreement: “Yes, both partners must want it, I think. […]. Otherwise, it won’t work. That is simply a fact. So, if, as soon as one party is forced to do it, […] at some point frustration will build up, which then can’t be handled anymore” (reference from participant 7 M, M for male).

#### Desire to implement the 50/50-split-model was a criterion in partner selection

The foundation of mutual agreement can be positively influenced via partner selection. Of the participants, 10 spoke of having selected a partner who also wants to implement the 50/50-split-model (R2). This is in line with reports by women in dual-earner relationships of another study [71]. In the present study, there were three participants who explicitly stated that they did not yet think about the 50/50-split-model when selecting their partner (R3). Other participants were ambivalent (R4) or did not speak about their partner selection at all. As attitudes can change [72], it is possible that the partners attitudes adapted since the beginning of the relationship. At the same time, the probability to seek a partner with similar role attitudes is high [73]. Therefore, attitudes of the respective partners may have been similar all along, even though they did not consciously select a partner with similar attitudes. In sum, selecting a partner who wants to implement the 50/50-split-model seems to be helpful for finding mutual agreement, but it also seems to be possible to find agreement later in the relationship. Once mutual agreement is reached, the following success strategies can be applied.

R2 Subcategory 1.2.1 Relevant criterion: “Well, like, how well you can assess it beforehand. But I think, if I would have had the feeling that [partner] wants to make a steep career and expects me to bring two to three children into the world and raise them, I think I would have run away screaming” (reference from participant 9 F, F for female).
R3 Subcategory 1.2.3 No relevant criterion: “When you’re 17, I don’t think you pay much attention to whether the man is helping out around the house” (4 F).
R4 Subcategory 1.2.2 Ambivalent: “Subconsciously perhaps. Yes, we were young (laughs), you don’t pay attention to things like that. You pay more attention to external features (laughs). Tricky” (17 M).

### Daily routine management

**Fig. 3:**
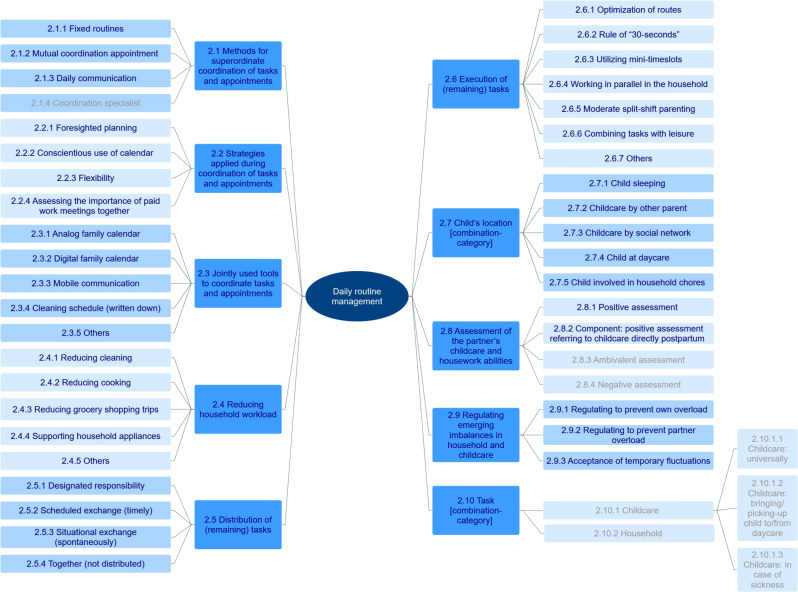
Daily routine management. Hierarchical level of main- and subcategories presented from inside (higher level) to outside (lower level). Subcategories are success strategies. Subcategories not counted as success strategies are *coordination specialist*, *ambivalent*, *negative assessment of the partner’s childcare and housework abilities*, and all subcategories of the combination-category task

The analysis revealed 38 practicable success strategies^1^ regarding daily routine management which parents can apply to implement a 50/50-split-model (Fig. 1F)^2^. On average, an individual participant applied 23 success strategies (SD = 3.83; range = 18–31). Each *subcategory* of the coding frame (Table 2) equates to one success strategy presented in italic font in the text. For an overview, the hierarchical level of the main- and subcategories/success strategies is presented in Fig. 3. The main category 2.10 is a combination-category and will be discussed in combination with the main categories 2.5, 2.7, and 2.8.

#### Methods for superordinate coordination of tasks and appointments

Family life with its different activities such as driving children to daycare, doctor’s appointments, or grocery shopping must be coordinated. Almost all participants used *fixed routines* to structure their days, weeks, or months, i.e., certain appointments or tasks were repeated in a fixed rhythm. This included, e.g., a fixed cleaning schedule or day, or regular weekly appointments (R5). Fixed routines can reduce the large amount of time and effort dual-earner parents spend on scheduling [49]. Additionally, nine participants had a regular *mutual coordination appointment*, i.e., a regular meeting during which the upcoming tasks and appointments were planned together. In most cases, the meeting was held weekly (R6). Moreover, almost all participants *communicated daily* and casually about the organization of tasks, e.g., during mealtimes or via phone (R7). A mutual coordination appointment and daily communication were also reported by other dual-earner parents [48, 49]. The difference between 50/50-split-model-parents and other dual-earner parents is that in most 50/50-split-model cases, both parents were responsible for the coordination appointment and daily communication. E.g., it was also the fathers who called the mothers to coordinate pick-up times (R7), whereas in other dual-earner parents, the mothers were primarily responsible for coordination [48, 49]. In our sample however, only seven 50/50-split-model participants had a *coordination specialist*, i.e., one responsible partner for the coordination of appointments and tasks (R8), in most cases the mother. This is potentially problematic, because coordination is an invisible task, part of the mental labor dimension “planning and strategizing” [74]. Mental labor is usually performed by mothers, which can have negative consequences for them, e.g., feelings of parenting role overload [74, 75]. Thus, parents designating coordination to one partner need to make this invisible task visible and designate a task of comparable effort to the other partner to reduce negative consequences. As this generally seems to be difficult, we are not recommending having a coordination specialist, unless parents are well reflected about the invisibility of coordination. In sum, for a successful implementation of the 50/50-split-model we recommend having fixed routines for appointments and tasks, discussing the weekly tasks during a mutual coordination appointment, and communicating daily. All strategies can be and are combined by the participants.

R5 Subcategory/success strategy 2.1.1 Fixed routines: “So, generally, it’s just important for our week that we have a plan. That we know: Okay, every Monday it’s the kitchen’s turn to get cleaned up” (20 F).
R6 Subcategory/success strategy 2.1.2 Mutual coordination appointment: “We actually try to get together on the weekend, usually on Sunday evening. And simply go over who has which plans for the week. And how we can best divide our tasks” (14 F).
R7 Subcategory/success strategy 2.1.3 Daily communication & example of a father being responsible for daily communication: “So, because my wife also works in a clinic and there can be new samples in the laboratory relatively short term, so to say. It can also happen that she can NOT pick-up the little one on time. And then I always reassure myself: ‘So, does it stay that way [that you can pick her up], is everything good, and so on?’ I actually do that [call her] every working day” (15 M).
R8 Subcategory 2.1.4 Coordination specialist (not counted as success strategy): “Mostly I (laughs). Because I actually make all the appointments. At least for the kids. […] I make sure that all the appointments are entered [into the calendar]. […]. But he’s also quite happy that I’m doing it. And that he doesn’t have to coordinate it” (11 F).

#### Strategies applied during coordination of tasks and appointments

During the coordination of tasks and appointments with the methods discussed above, certain strategies can be applied. E.g., while holding the mutual coordination appointment, parents can plan foresightedly and use their calendar conscientiously. During daily communication, flexible solutions for unforeseen events can be found.

Almost all participants *planned and acted foresightedly*. This included, e.g., having emergency plans, asking for childcare assistance in advance of business trips, or discussing upcoming routine changes (R9). About half of the participants spoke of using their *calendar in a conscientious manner*, which included writing down appointments “always” or “directly”, prioritizing appointments which were written down, and checking calendars before making new appointments (R10). All participants showed *flexibility*, i.e., could respond adaptively to unplanned changes, e.g., child sickness or last-minute appointments. They could re-coordinate planned routines on short notice and find alternative solutions (R11). In situations, where work meetings conflicted with child-related obligations, participants *assessed the importance of paid work meetings* together. The couples discussed whose meeting was more dispensable and who could thus leave work to care for the child. Participants often evaluated internal team meetings as less important than external meetings (R12). Such discussions have also been reported by other dual-earner parents [49, 52] and are necessary to not slip into a pattern where one partner always thinks they have the more important work meeting.

Interestingly, planning foresightedly and flexibility were often spoken about in close proximity to one another. Participants planned foresightedly but had to remain flexible if something came up (R13). The same was true for fixed routines and flexibility (R14). Thus, participants combined seemingly opposing strategies: a certain rigor with situationally necessary flexibility. It is difficult to compare this behavior to other dual-earner parents, as “family management” is often not further broken down into specific strategies (e.g., [48, 76]).

R9 Subcategory/success strategy 2.2.1 Foresighted planning: “Well, I think some objects just have to be there twice. So, e.g., that we have a lot of things at grandma’s house, too. […] E.g., just a car seat and a bike seat at grandmas. That there’s not so much organizational stuff then/ That [grandma] can also spontaneously pick [child] up if something came up” (13 F).
R10 Subcategory/success strategy 2.2.2 Conscientious use of calendar: “And really all appointments are entered in there. And it updates itself automatically when you’re on the WLAN. And we’re actually relatively strict about that, because the appointments that aren’t in there can’t be taken into account” (25 F).
R11 Subcategory/success strategy 2.2.3 Flexibility: “I work with a colleague and because we share the work, we can also switch around a bit. So, if something comes up, she takes over the 4:45 p.m. shift, so to say” (10 M).
R12 Subcategory/success strategy 2.2.4 Assessing the importance of paid work meetings together: “Then we assess which appointment is more dispensable. […] Once a month I have a meeting Tuesday afternoon. […]. There is a protocol for this meeting. And if my husband has another important appointment during that time […] then I can, phew (indifferent), painlessly do without it. But of course, there are also project meetings when we have big events. […]. Then we coordinate this on the phone at short notice: ‘And what is your deadline? What is my deadline? Can you perhaps participate [in your meeting] over a conference call? Do I have the option of sending someone else to report to me?’ That’s really a question of tradeoff then” (20 F).
R13 Example of subcategories foresighted planning and flexibility in close proximity to one another: “Well, we have contingency plans, as they say (laughs). So, if we can’t organize it, grandma will gladly step in, yes. […] It all has to be organized beforehand. And not overnight. Sometimes it takes weeks to plan how we’re going to organize it [*foresighted planning*]. I always think it’s really bad when they cancel on short notice. […] We just had this problem last week when my husband was scheduled for surgery. And the little one was sick. And grandma said, ‘Yes, okay, I’ll watch her’. And she wasn’t there, she had overslept. And we had to organize and do something very quickly. And in five minutes we changed everything, our entire plan. We quickly got the child dressed. We drove the child to daycare. And my husband was still punctually at the operation at 7:00 a.m. so that we could get started [*flexibility*]” (11 F).
R14 Example of subcategories fixed routines and flexibility in close proximity to one another: “Yes, we stick to the [household-] plan [*fixed routines*], but if once it is just not [doable] today, then I do it tomorrow [*flexibility*]” (5 M).

#### Jointly used tools to coordinate tasks and appointments

The participants used multiple coordination tools together, most frequently an *analog family calendar* (17 participants). Some participants used a *digital family calendar* (nine participants). Of those, four participants used both an analog and a digital family calendar, meaning that three participants used neither. For both calendar types, participants spoke of advantages (R15–16). Dual-earner and traditional families also use family calendars. Unique for the majority of our 50/50-split-model-parents is the high involvement of both parents in the calendaring routines ([77]; in some 50/50-split-model-families, mothers are still the coordination specialist [see 2.1] and primary scheduler). *Mobile communication*, such as phone calls or messenger services to coordinate daily tasks, was mentioned by 12 participants (R17). This is surprisingly low, considering that 90% of Germans use a cellphone [78]. A potential explanation is that the usage of mobile communication for coordination purposes decreases with longer planning horizons [79] and 50/50-split-model-parents plan foresightedly. Nonetheless, we expected a higher use of mobile communication than the reported numbers. Therefore, we assume that mobile communication was not reported by the remaining 13 participants, as they did not perceive it as a very important coordination tool for the success of the 50/50-split-model. A *written down cleaning schedule* was reported by six participants (R18). *Other coordination tools*, such as to-do-lists or organization apps were mentioned by three participants. The most important jointly used coordination tool seems to be the analog or digital family calendar, depending on the preferences of the participants.

R15 Subcategory/success strategy 2.3.1 Analog family calendar: “It hangs directly in the living room, next to the dining table. So, yes, that you actually see it at breakfast, at supper, always while eating, you look at it and then you know right away: ‘Ah, I still have to write something down’” (1 M).
R16 Subcategory/success strategy 2.3.2 Digital family calendar: “And what we’ve been doing now lately: We have actually set up the possibility that we can view each other’s calendars that we both keep, the digital ones. That means I can also, if I somehow MUST schedule a work-related appointment or juggle a bit, I can also see which tasks [partner] somehow currently has. Or rather appointments. And accordingly, already ahead of time somehow/ So not, somehow, first create appointment conflicts and then say afterwards: ‘Oh, you have one, too/’. It’s actually quite practical. You can look a bit and, in case, take action” (22 M).
R17 Subcategory/success strategy 2.3.3 Mobile communication: “We communicate a lot. There’s a lot/ So, of course we’re now also using all the new media like Facebook, WhatsApp and so on. If something comes up at short notice, we always write: ‘Here, I’m going to be a bit late. Can you pick up the little one?’” (2 M).
R18 Subcategory/success strategy 2.3.4 Cleaning schedule (written down): “So, we have a fixed plan, and it’s written down when to do which laundry. Or when the beds need to be changed again” (20 F).

#### Reducing household workload

Participants reduced their amount of household workload by omitting chores or using support. All participants reported to *reduce cleaning* in multiple references (66 references; R19). Other strategies included *reducing cooking*, e.g., by having bread for dinner (R20), and *reducing grocery shopping trips*, i.e., doing one big shopping trip per week (R21). Utilizing *supporting household appliances*, such as a thermomixer or dishwasher, was reported by 16 participants (R22). None of the participants hired paid help to reduce household workload. Previous studies showed that “leaving some things undone around the house” can help dual-earner parents cope with work-family role overload [80], underlining the importance of these success strategies. Moreover, the participants reported that reducing household workload created time for childcare and leisure, despite both parents participating in paid work (R19).

R19 Subcategory/success strategy 2.4.1 Reducing cleaning & example of how this creates time for childcare and leisure: “So, you can see it in multiple corners. So you see, e.g.,/ You could have, e.g., before you decorate windows, you could have cleaned windows. COULD have been done. We did NOT. So, because then I told myself, ‘What’s more important?’ And then I said, ‘It’s the time with the child. And then the windows stay like that’” (21 F).
R20 Subcategory/success strategy 2.4.2 Reducing cooking: “Otherwise we, well, we tend to cook on the weekend. During the week it usually has to be quick. So sometimes we just have a sausage (laughs) or something like that” (19 F).
R21 Subcategory/success strategy 2.4.3 Reducing grocery shopping trips: “I used to do that when I was living […] without a child, when we needed something, we would go out again quickly in the evening. […] And now we’re really trying to write a shopping list. The old-fashioned way. And really buy as much as possible. […] we definitely try to save time with that” (6 M).
R22 Subcategory/success strategy 2.4.4 Supporting household appliances: “Finally a dishwasher (laughs). So that you no longer have to wash up by hand. And a big one, so you don’t have to wash pans and other things by hand. So, I think that’s also something that saves us time. That we now simply have a large dishwasher” (12 M).

#### Distribution of (remaining) tasks

The household and childcare tasks remaining after reducing household workload must be distributed between the parents. Most participants applied a mixture of different distribution mechanisms, depending on the type of task (Table 3). *Childcare tasks (universally)* include all direct contact with the child, such as feeding, playing, or family trips, but not *bringing/picking-up child to/from daycare* and *childcare in case of sickness*, which are represented in separate subcategories. *Household tasks* include all household related tasks, i.e., routine housework, such as laundry, cooking, or cleaning, but also organizational tasks, such as superordinate coordination or filing tax returns.

**Table 3:** Distribution of (remaining) t﻿asks combined with tasks

		Task			
		Childcare: universally ^a^	Childcare: bringing/picking-up child to/from daycare	Childcare: in case of sickness	Household
**Distribution of (remaining) tasks**	Designated responsibility	17 (49)	12 (30)	9 (12)	23 (131)
	Scheduled exchange (timely)	13 (24)	14 (41)	8 (10)	9 (13)
	Situational exchange (spontaneously)	19 (49)	6 (8)	10 (16)	17 (53)
	Together (not distributed)	21 (52)	4 (4)	2 (2)	20 (51)

*Note* Participants (number of references)

^a^ Includes all childcare tasks except bringing/picking-up child to/from daycare and childcare in case of sickness

*Designated responsibility* means that one partner was always responsible for one certain task. This distribution mechanism was often used for routine housework tasks (R23). In high-income countries, routine, time-consuming housework is usually done by mothers [81]. In our sample, fathers also took on designated responsibility for routine housework (R23). Often, participants stated that tasks were designated according to preference or skill (R23). This might contribute to feeling fulfilled while doing housework, which has previously been found to have a significant positive impact on an equal distribution of housework [58]. Bringing/picking-up child to/from daycare was often *exchanged by a regular schedule*, meaning that tasks were switched rhythmically, e.g., each second day (R24; Table 3).

Both designated responsibility and regular exchange schedules have two advantages. Firstly, they can relieve mothers of the mental responsibility for these tasks. In dual-earner but not 50/50-split-model populations, it has been shown that even if fathers pick-up their children sometimes, mothers remain responsible for the organization of the pick-up [82]. With a designated responsibility or regular exchange schedule the mental responsibility can also be exchanged (R24–25). The second advantage is that designated responsibility and regular exchange schedules can build up confidence and self-efficacy in fathers. E.g., if a father has the designated responsibility for bathing the child by himself, he can build up self-efficacy regarding this task, which in turn can reinforce his involvement and the 50/50-split-model (R26; [51, 83, 84]).

*Situational exchange* of universal childcare tasks was typically applied if one partner was tired or stressed. Then the other took over (R27). This has previously been reported as a strategy that can relieve parenting stress [51]. Situational exchange was also often used for childcare in case of sickness (R28; Table 3) and by participants who had high flexibility requirements at their workplace (e.g., shift work; R29). Lastly, participants did many childcare tasks *together*, such as playing, bedtime routine, or activities (R30; Table 3). Some housework tasks were also done together, typically going grocery shopping and sometimes laundry (R31). With this mixture of different distribution mechanisms, participants seemed to have enough structure to be able to uphold an equal distribution, but also sufficient flexibility to react situationally, e.g., if one partner needed a break, had to work, or the child got sick.

R23 Subcategory/success strategy 1.5.1 Designated responsibility combined with 1.10.2 Household & example for fathers taking designated responsibility for routine housework & example for tasks being designated according to preference or skill: “Because that’s also relatively clearly distributed. So, e.g., I hate doing anything in the kitchen. Whether it’s cleaning up the kitchen or loading or unloading the dishwasher. And so that’s really one of my husband’s fixed chores. And he finds things like dusting or just tidying up the rooms, tidying up the kids’ room, he says, ‘Those are terribly sucky chores’. But I find that quite, yes, quite relaxed” (20 F).
R24 Subcategory/success strategy 2.5.2 Scheduled exchange (timely) combined with 2.10.1.2 Childcare: bringing/picking-up child to/from daycare & example for scheduled exchange relieving mothers of the mental responsibility for tasks: “It’s like, on Mondays I bring the little one away, she picks him up. On Tuesdays she brings him away, I pick him up. That’s all fixed for the next few weeks” (17 M).
R25 Subcategory/success strategy 2.5.1 Designated responsibility combined with 2.10.1.2 Childcare: bringing/picking-up child to/from daycare & example for designated responsibility relieving mothers of the mental responsibility for tasks: “Then I drive to work. And he waits at home until the little one wakes up. And I guess she always wakes up around 7:00/7:30. Then he/ And then he has breakfast with her. And drives her to the nursery, so that she is in the nursery around, between 8:00/8:30. Then he takes the train to work. And I pick up/ have to work until 3:30 and then pick up the little one around 4:00 from the nursery again” (19 F).
R26 Subcategory/success strategy 2.5.1 Designated responsibility combined with 2.10.1.1 Childcare: universally & example of father building up self-efficacy regarding this task:

- Female partners interview: “Until at some point my husband said, ‘You can go take a shower now. I’ll do the little one’. I was like, ‘Okay’. And then I was like: ‘You’re not even going to look in the nursery now HOW he’s doing it. The kid’s not screaming, so it’ll be fine’. Like that. And so, each has found their own” (21 F).
- Male partners interview: “So, in the beginning it was ONLY [wife] who put [child] to bed. I somehow didn’t trust myself to do that or something. Like, that was not my thing. Then I had to do it at some point, because there was no other way and it worked. So then, that was out of the way” (10 M).

R27 Subcategory/success strategy 2.5.3 Situational exchange (spontaneously) combined with 2.10.1.1 Childcare: universally: “Exactly, when it comes to childcare, it’s more or less the case that we ask it of each other. That we sometimes say: ‘Now I need a bit of rest, you do something with the child’” (12 M).
R28 Subcategory/success strategy 2.5.3 Situational exchange (spontaneously) combined with 2.10.1.3 Childcare: in case of sickness: “During that week [of child’s sickness], I had a lot to do at work, because things had to be finished. Of course, if it would have been really bad, I could have taken time off. But at [partners’] workplace it was less important. That’s why he said he’d do it. […] But that was a bit dependent on work” (13 F).
R29 Subcategory/success strategy 2.5.3 Situational exchange (spontaneously) combined with 2.10.1.2 Childcare: bringing/picking-up child to/from daycare by participants who had high flexibility requirements at their workplace: “And then I pick up [child] from childcare. Or [partner] picks him up, depending on what shift he has” (23 F).
R30 Subcategory/success strategy 2.5.4 Together (not distributed) combined with 2.10.1.1 Childcare: universally: “Or just to baby swimming. It went on for […] eight weeks. Sunday morning, we had to get up at 06:00, so that we were at the swimming pool at 07:30 on a Sunday. The father of my friend’s child was there the first time but not the other seven times, because it was too early in the morning for him. Whereas my partner says, that’s not even a question for HIM. Because that is time, which he can spend with me, which he can spend with [child]. And especially the baby swimming, these are such events and such moments, which he WON’T miss with [child]” (23 F).
R31 Subcategory/success strategy 2.5.4 Together (not distributed) combined with 2.10.2 Household: “Well actually we do the laundry on weekends, also together, that we throw a big pile in the hallway and the little one can join” (4 F).

#### Execution of (remaining) tasks

In addition to the option of reducing household workload by omitting certain tasks (see 2.4), techniques of how tasks are executed offer further opportunities to save time and maintain the 50/50-split. The strategy mentioned most frequently was *optimization of routes*. This included doing things on the way home, e.g., buying fresh bread, or choosing a daycare facility close to home (R32). Routes have been identified as extremely time consuming for families [85], making this an important success strategy for the 50/50-split-model. The *rule of 30-seconds* is an in-vivo category mentioned by one participant: “*And then we introduced a 30-second rule. […] My husband came up with that. Everything that can be done within 30 seconds is done immediately. So*, e.g.,* if you’ve just drunk a glass of water*,* you put it straight into the dishwasher instead of on top of it (laughs). […] And then the housework issue no longer plays such a big role.*” Interestingly, even though not calling it “rule of 30-seconds”, nine more participants mentioned cleaning up things right away to prevent a big buildup of chores taking a long time to complete. *Utilizing mini-timeslots* differentiates from that, as chores were not done right away, but if short, unplanned windows of time arose, e.g., if one still had 10 min left before one had to leave the house (R33). Another frequently reported strategy was *working in parallel in the household*, often emphasized as important to maintain the 50/50-split (R34).

Many participants used *moderate split-shift parenting* regarding bringing/picking-up child to/from daycare. Parent A brought the child (later) while parent B went to work (earlier). In the afternoon parent B picked up the child (earlier) while A could stay at work (longer). This helped to fulfil work related requirements, reduced daycare time for the child, and increased parent-child time (R35). Moderate split-shift parenting has previously been reported by other dual-earner parents [52]. Emphasizing “moderate”, most participants shifted their working times for a few hours. This is not to be confused with split-shift parenting, which is a term used if parents shift whole days, with one parent working evenings, nights, or weekends [86, 87]. Split-shift parenting is associated with the missing availability of formal childcare, i.e., parents having no other choice [86] and not recommended in this study. We do not expect parents to undertake exorbitant efforts on the one side while politics do not provide necessary work-family structures on the other side. While focusing on behavioral strategies parents can utilize to implement a 50/50-split-model in this study, we generally argue that behavioral and environmental determinants interplay in attaining gender equality (see Conclusion).

*Combining tasks with leisure*, e.g., watching TV while ironing, was mentioned by five participants (R36). Other dual-earner parents also combine unpaid work with leisure and this combination is associated with emotional well-being. This has similar positive outcomes as pure leisure time [88]. Taken together, these strategies can create time for childcare and leisure, despite both parents participating in paid work.

R32 Subcategory/success strategy 2.6.1 Optimization of routes: “And then I usually go shopping with the little one. On the way home [from the daycare center], we pass by the supermarket” (8 M).
R33 Subcategory/success strategy 2.6.3 Utilizing mini-timeslots: “So, if you have a free minute, you just use it to tidy up quickly” (16 F).
R34 Subcategory/success strategy 2.6.4 Working in parallel in the household: “And when the little one takes a nap, we have a rule that if one of us does something around the house, the other one does something too” (21 F).
R35 Subcategory/success strategy 2.6.5 Moderate split-shift parenting: “One of us takes him to the daycare and the other one can, like, go to work half an hour earlier or an hour […]. So the one who brings him in the morning can stay longer at work [in the afternoon]. […] [And that] is really switched day by day. Because otherwise, it wouldn’t be possible to manage with the hours. So that’s like 38 hours a week public service and, yes, otherwise it would not be possible with the daycare times. Or well, it would be possible, but you wouldn’t see your child” (7 M).
R36 Subcategory/success strategy 2.6.6 Combining tasks with leisure: “And I actually hang up the laundry mostly in the evenings. It’s really become a time of relaxation. Then I turn on some YouTube video or a podcast and then hang up the laundry” (3 F).

#### Child’s location during the execution of household tasks

Table 4 shows that the child was often *sleeping* during parents’ execution of household tasks, i.e., household tasks were completed in the mornings, evenings, or during naptime (R37). Equally important was the technique of one parent executing household tasks, while the *other was looking after the child* (R38). Even though still young, children were often already *involved in household chores*, e.g., parents were taking them for grocery shopping or encouraging to help with easy tasks. Involving children was reported as enjoyable for both parents and children (R39). Other dual-earner parents also combine housework with childcare [89]. Childcare by *social network* and *daycare* played a minor role for the execution of household tasks, except for participants with high flexibility requirements at their workplace (e.g., shift work; R40). None of the participants hired paid help for childcare.

**Table 4:** Child’s location comb﻿ined with household ta﻿sks

		Task
		Household
**Child’s location**	2.7.1 Child sleeping	20 (53)
	2.7.2 Childcare by other parent	19 (55)
	2.7.3 Childcare by social network	4 (6)
	2.7.4 Child at daycare	7 (16)
	2.7.5 Child involved in household chores	21 (50)

*Note* Participants (number of references)

R37 Subcategory/success strategy 2.7.1 Child sleeping combined with 2.10.2 Household: “[…] put the child down, to sleep. Then most times it is 7/7:30 p.m. Yes, and then you have FREE TIME. Then you have to […] restore a basic order then. For example, kitchen or playroom. Maybe another half/three-quarters of an hour and then, yes, spend the evening comfortably” (7 M).
R38 Subcategory/success strategy 2.7.2 Childcare by other parent combined with 2.10.2 Household: “Now it’s usually the case that one of us cooks and one of us looks after our son, of course. That he’s not crawling around under the stove (laughs)” (16 F).
R39 Subcategory/success strategy 2.7.5 Child involved in household chores combined with 2.10.2 Household & example for this time being enjoyable for both parents and children: “Or to vacuum the apartment. The little one (laughs) is always happy when the vacuum cleaner goes on. He always sits next to it and is fascinated by the noise and how it all works. And then he runs after it, joyfully jumping” (2 M).
R40 Subcategory/success strategy 2.7.4 Child at daycare combined with 2.10.2 Household by participants with high flexibility requirements at their workplace: “Yes, although of course it also has advantages, if you have the late shift, you really have a morning for yourself. That is not so bad, to be able to do appointments. And also do the household during that time” (25 F).

#### Assessment of the partner’s childcare and housework abilities

Beyond the more “hands-on” strategies of the preceding categories, *a positive assessment of the partner’s childcare and housework abilities* also seems beneficial for the 50/50-split-model. All participants assessed the abilities of their partner positively (R41–42), especially regarding childcare (Table 5). Some positive assessments specifically referred to *childcare abilities directly postpartum* (R43). A positive assessment made it easier to trust and share tasks. There were around 80% less *negative* than positive statements about the partner’s childcare and housework abilities. Negative assessment is similar to the dimension “standards and responsibility” of maternal gatekeeping (R44; [90]). Greater maternal gatekeeping is associated with less paternal involvement in multiple studies [91, 92], whereas maternal perception of paternal competence is associated with more paternal involvement [51, 91]. Deutsch and Gaunt [71] have identified “anti-essentialism”, the belief that the father is just as capable in nurturing as the mother, as a key conviction in facilitating equality. The present results are thus in line with prior literature, all together strongly showing that a positive assessment of the partner’s abilities can help implement a 50/50-split-model.

**Table 5:** Assessment of the partner’s abilities combined with tasks

		Task	
		Childcare: all ^a^	Household
**Assessment** **of the partner’s** **abilities**	Positive assessment	24 (110)	20 (38)
	Component: positive assessment referring to childcare directly after birth	13 (16)	0 (0)
	Ambivalent assessment	10 (13)	7 (8)
	Negative assessment	6 (8)	10 (21)

*Note* Participants (number of references)

^a^ Includes all childcare tasks

R41 Subcategory/success strategy 2.8.1 Positive assessment combined with 2.10.1 Childcare: “And one of my colleagues also said: ‘Well, does your husband have the courage to do that and can he do it?’ Where I say: ‘Well, why not? He’s the father. What’s that all about? It’s also his child. Why shouldn’t he be able to do it?’ That is yes/ That was incomprehensible to me” (19 F).
R42 Subcategory/success strategy 2.8.1 Positive assessment combined with 2.10.2 Household: “At our house both of us iron, both of us do laundry, both of us clean. Like, there is ZERO difference. And everyone does it the same way, in my opinion. Or there’s no discussion, you didn’t do it right or you did it wrong or something” (24 F).
R43 Subcategory/success strategy 2.8.2 Component: positive assessment referring to childcare directly postpartum: “[…] we said from the beginning, ‘I’m breastfeeding. You’re diapering’. And yes, that’s the way it is. He was better at it in the beginning” (25 F).
R44 Subcategory 2.8.4 Negative assessment (not counted as success strategy) combined with 2.10.2 Household: “Or vacuuming. I’m very picky about that. He can’t do it/ not quite right for me. I prefer to do that myself” (19 F).

#### Regulating emerging imbalances in household and childcare

Despite all preceding strategies to manage the daily routine, sometimes inequalities in the distribution of tasks emerged. If participants perceived to be taking over more tasks themselves, they asked their partner to take over (*regulating to prevent own overload*; R45). This entailed first, to recognize own overload and second, ask for help. It was thus a form of self-care, which is the ability to “[…] regard one’s own needs, assess stresses correctly, remain sensitive to excessive demands, or not to overexert oneself [translated]” [93, p. 515]. If participants perceived their partner to be taking over more tasks, they took over themselves (*regulating to prevent partner overload*; R46). This entailed first, to recognize partner overload by being empathetic and second, willingness to help. Regulating emerging imbalances in this manner may be a form of reciprocal altruism [94], where the helper expected that their partner would help them in return in the future. If the expected help did not come in, they may have felt entitled to ask for it. In some cases, it was helpful to *accept temporary fluctuations* if the distribution of tasks was balanced on average. Accepting temporary fluctuations has previously been described as “goodwill” in parents with active paternal involvement in childcare [51]. This goodwill or trust that fluctuations will ultimately even out, was not exploited by the respective partners. All three strategies were often mentioned within proximity to each other, with an attentive eye for oneself, the partner, and the current situation. A balance between those seemingly opposing strategies seems to be necessary for the 50/50-split-model (R47–48).

R45 Subcategory/success strategy 2.9.1 Regulating to prevent own overload: “And THAT [standing up if the child wakes up early in the morning] is like sometimes, where we have had some arguments, when no one wanted and then I said: ‘No, now you have to go’” (24 F).
R46 Subcategory/success strategy 2.9.2 Regulating to prevent partner overload: “I think we notice that about each other. Like, then you just say, ‘You’ve done that, you’ve done that enough times now. Now it’s my turn again’. So, both partners are, I think, sensitized to the fact that you must make sure that it’s not a one-sided thing. But that it is shared” (18 M).
R47 Example of subcategories regulating to prevent own overload and regulating to prevent partner overload in close proximity to one another: “Rather casually, along the lines of: ‘Here, I’d do the dishes now. In the meantime, could you please take the paper trash downstairs’ [*regulating to prevent own overload*]. […]. And then the question came: ‘Man, I’m done now. I see you’re still busy doing the dishes for a few minutes, what should I do in the meantime?’ [*regulating to prevent partner overload*]. Yes, and so it just came from both sides” (21F).
R48 Example of subcategories acceptance of temporary fluctuations and regulating to prevent own overload in close proximity to one another: “In the trainee program, it was just this special situation, because it was basically a further education for my husband. And then I said: ‘Yes, that’s fine [*acceptance of temporary fluctuations*]. But when it’s over and you’re permanently employed in the company. Then I would like to have the same opportunity to develop myself again. And I don’t want to have the majority of the tasks, but I would like to have the same opportunity to develop further’. And I made that clear relatively early on [*regulating to prevent own overload*]” (20 F).

## Summary of results and discussion

To summarize, we will integrate our results into our theoretical framework (Fig. 1). Prior research found that if couples have equal time availability and resources, they are more likely to implement egalitarian gender role distributions (e.g., [29]). Moreover, individuals with more egalitarian values are more likely to implement egalitarian gender role distributions (e.g., [29]; Fig. 1A). In line with this latter gender ideology theory, our results revealed that almost all participants had a mutual agreement to implement an egalitarian gender role distribution. Couples’ being set-to-equal in these areas, thus, seems like a necessary foundation to be able to implement a 50/50-split-model, where parents share paid work, childcare, and housework equally between each other (Fig. 1B). Our results show that some participants influenced the necessary mutual agreement via partner selection.

After being set-to-equal, macro-level predictors, individual-level predictors, gender display (e.g., [29]), and behavioral-level predictors could all play a role in being able to implement a 50/50-split-model (Fig. 1C–F). This QCA focused on behavioral-level predictors and revealed a catalog of 38 practicable strategies to manage daily routine^3^, which can help parents to successfully implement the 50/50-split-model (Fig. 1F). To coordinate daily routine, it is helpful to define fixed routines, have a weekly coordination appointment, and communicate daily. While organizing, it is helpful to plan foresightedly. At the same time, unpredicted changes should be met with flexibility. Moreover, it is helpful to assess the importance of paid work meetings together. An important coordination tool is the family calendar, which should be used conscientiously. Next to the higher-level coordination of daily life, childcare and household tasks must be executed, which can be time consuming. It is thus helpful to reduce cleaning, cooking, and grocery shopping, and to use supporting household appliances. All remaining tasks can be distributed with an established structure, e.g., designating responsibility for tasks, or a regular exchange schedule. At the same time, it is helpful to sometimes loosen those established structures and exchange tasks situationally, e.g., if one partner is tired. Moreover, it can be enriching for family life to execute certain tasks together. After distribution, there are some helpful strategies for the execution of tasks, including optimization of routes, the rule of 30-seconds, utilizing mini-timeslots, working in parallel in the household, moderate split-shift parenting, and combining tasks with leisure. Household tasks can be executed while the child is sleeping, taken care of by the other parent, or the child can be involved in household tasks. Regarding interpersonal success strategies, it is helpful to positively assess the partner’s childcare and housework abilities, also early postpartum. If, despite all these success strategies, imbalances occur, it is important to recognize these and either prevent own or partner overload by requesting or providing assistance. Preventing partner overload might even be helpful to reduce unwanted, subconscious gender display (e.g., [29]), as partners look out for each other.

It becomes apparent that a combination of seemingly opposing strategies is advantageous for the 50/50-split-model. Foresighted planning was combined with flexibility, regular with situational exchange of tasks, and preventing own with preventing partner overload. The seemingly opposing strategies were well balanced by the participants, which might be unique for 50/50-split-model-parents. The list of success strategies is thus a catalog of strategies, meaning multiple, but not all strategies must be applied for success. On the one hand, by utilizing one success strategy only, parents will probably not achieve a 50/50-split-model. On the other hand, if parents are planning to implement the 50/50-split-model, they can resort to a selection of success strategies that appeal to them. E.g., it might not be appealing for all parents to reduce cooking, because some might find it important to eat a warm meal in the evening. Those parents can resort to other success strategies to reduce their workload, e.g., reduce cleaning. Of the 38 success strategies regarding daily routine management^4^, participants used on average 23.

Some of our behavioral-level success strategies might interplay with prior theories [46, 47]. Macro-level predictors such as culture might influence how much a couple communicates daily or the extent to which they are willing to reduce their cleaning (Fig. 1C). Individual-level predictors such as a person’s learning experiences might influence how flexible they are or how they assess their partner’s childcare and housework abilities (Fig. 1E). Nonetheless, (new) behavior can be learned [95] and thus we posit that our behavioral-level strategies can be independent of external circumstances (Fig. 1F).

### Limitations and research implications

The present sample consists of white parents with above average incomes. Additionally, the sample consists of parents who were already attempting to achieve a 50/50-split-model. The 38 success strategies have not yet been tested to determine their efficacy in assisting others to implement a 50/50-split-model. Thus, it is important to be careful to generalize the findings to other populations. Nonetheless, it was our aim to find practicable strategies on a behavioral-level which new parents can apply in their daily routine to successfully implement the 50/50-split-model if they wish to do so. Those are relatively independent of external circumstances. Thus, we invite all parents who wish to implement the model to select those success strategies from the catalog that appeal to them and are relevant for their individual situation. Additionally, future research could test how effective the strategies are in different populations to implement a 50/50-split-model.

This research is cross-sectional. Thus, we can only show strategies that help parents to implement the 50/50-split-model at 17 months postpartum. It would be interesting to conduct a follow-up study with the population, to research whether the parents were able to uphold the 50/50-split-model over longer durations of time.

Our focus on behavioral-level strategies is a strength, but also a limitation. Parents are not solely responsible for achieving a 50/50-split-model and environmental predictors, e.g., politics, undoubtedly play a role as well, as prior research has shown. In our study, we presented a theoretical framework of how our success strategies might interplay with prior research, but it was not our aim to test this framework. Future studies could investigate how our success strategies interplay with prior theories and potentially confirm our proposed theoretical framework of Fig. 1.

## Conclusion

We purposively sampled an exclusive group of egalitarian parents implementing a 50/50-split-model 17 months postpartum. This means, these parents were sharing the domains paid work, childcare, and housework equally between each other. This group is unique, because usually, even dual-earner parents share these domains unequally [3–6]. For parents who wish to implement the 50/50-split-model, this study aimed to find practicable strategies on a behavioral-level which they can apply in their daily routine to do so. With problem-centered interviews [62] analyzed via QCA [67] we found an extensive list of 38 practicable success strategies which new parents can apply to implement the 50/50-split-model. Additional file 1 provides a list of the 38 strategies with a brief description of what each strategy entails. Couples, who wish to implement the 50/50-split-model, can consult the list for a quick overview.

Some studies have previously identified some of the success strategies, rendering our research valid (e.g., [48, 50, 51]). But, to the best of our knowledge, we are the first to identify such an extensive list of practicable success strategies on the behavioral-level. Moreover, our success strategies are concerning the daily routine and therefore relatively independent of external circumstances. They are thus a low-threshold gateway for parents who wish to implement a 50/50-split-model. This extends previous literature, which developed theories for persisting gender role inequalities predominantly focusing on circumstances and external predictors (for reviews, see [29–31]). These theories undoubtedly have explanatory power, but they do not help individuals to push towards gender equality (compare [41]). On the premise that “human functioning is a product of a reciprocal interplay of intrapersonal, behavioral, and environmental determinants” [46 p. 165, 47] both approaches—theories of environmental determinants and behavioral success strategies—have their justification. As shown in Fig. 1, we believe that prior theories and here presented success strategies interplay to finally achieve gender equality at work and at home.

In sum, we found a catalog of behavioral-level success strategies which can help parents implement a 50/50-split-model. This catalog can assist more parents to pioneer in implementing the model. This may lead to a healthier and happier public population, seeing prior research has shown that a 50/50-split-model might be beneficial for mental health, physical health, and relationship satisfaction [15–26].

### Electronic supplementary material

Below is the link to the electronic supplementary material.

Supplementary Material 1

## Data Availability

The data presented in this article is not publicly available because of legal and ethical constraints established by the Ethics Committee of the Faculty of Medicine of the Technische Universität Dresden. Requests to access the datasets should be directed to the project manager and principal investigator Susan Garthus-Niegel (contact via susan.garthus-niegel@ukdd.de).

## Footnotes

1 Subcategories not counted as success strategies: *Coordination specialist*, *ambivalent*, *negative assessment of the partner’s childcare and housework abilities*, and all subcategories of the combination-category task.

2 Some success strategies might not exclusively be applied by parents implementing a 50/50-split-model and might also be helpful to organize family life in more traditional families. This work does not aspire to present exclusive, but helpful strategies.

3 Subcategories not counted as success strategies: *Coordination specialist*, *ambivalent*, *negative assessment of the partner’s childcare and housework abilities*, and all subcategories of the combination-category task.

4 Subcategories not counted as success strategies: *Coordination specialist*, *ambivalent*, *negative assessment of the partner’s childcare and housework abilities*, and all subcategories of the combination-category task.

## Abbreviations

DREAM: DResdner Studie zu Elternschaft, Arbeit und Mentaler Gesundheit; Dresden Study on Parenting Work, and Mental Health
QCA: Qualitative content analysis
R: Sample reference
F: Sample reference from female participant
M: Sample reference from male participant
