# Supplementary material for: Practicable strategies parents can apply in their daily routine to successfully implement the 50/50-split-model of paid work, childcare, and housework: a qualitative content analysis
Source: BMC Public Health. 2024 Aug 14;24:2215. doi: 10.1186/s12889-024-19646-9 (PMC11323602; doi:10.1186/s12889-024-19646-9)
Supplement: Supplementary file 1 — Supplementary Material 1 [file 12889_2024_19646_MOESM1_ESM.pdf]

Additional file 1: List of 38 strategies in the daily routine to successfully implement the 50/50-split-model

Dear parents, if you wish to implement an equal split of paid work, childcare, and housework between each other, you can consult this list. It provides a quick overview of strategies you can apply in your daily routine to help you implement the 50/50-split-model. Please note that you do not have to apply all strategies for success but can resort to a selection of strategies that appeal to you. E.g., it might not be appealing for you to reduce cooking, because you might find it important to eat a warm meal in the evening. If this is the case, you can resort to other success strategies to reduce your workload, e.g., reduce cleaning.

Please refer to the main article for more detailed information on the strategies and sample references from participants, who have applied the strategies.

**Success strategy**	**Description**
**2.1 Methods for superordinate coordination of tasks and appointments**	
2.1.1 Fixed routines	Structure your days, weeks, or months with fixed rhythms. E.g., have a fixed cleaning schedule or day, or regular weekly appointments.
2.1.2 Mutual coordination appointment	Have a regular meeting with your partner, during which you plan your upcoming tasks and appointments, e.g., every Sunday evening.
2.1.3 Daily communication	Additionally, communicate daily and casually about the organization of tasks, e.g., during mealtimes or via phone. Both parents should feel responsible for daily communication.
**2.2 Strategies applied during coordination of tasks and appointments**	
2.2.1 Foresighted planning	Plan and act foresightedly, e.g., have contingency plans, ask for childcare assistance in advance of business trips, discuss upcoming routine changes, or plan your times realistically.
2.2.2 Conscientious use of calendar	Always and directly write down appointments, check calendars before making new appointments, and, in case of accidental double-bookings, prioritize appointments which were written down.
2.2.3 Flexibility	Respond adaptively to unplanned changes, e.g., child sickness or last-minute appointments. Re-coordinate planned routines on short notice and find alternative solutions.
2.2.4 Assessing the importance of paid work meetings together	If both you and your partner’s paid work meetings conflict with child-related obligations, discuss together whose meeting is more dispensable and who can thus leave work to care for the child. E.g., you might evaluate internal team meetings as less important than external meetings.

**2.3 Jointly used tools to coordinate tasks and appointments**	
2.3.1 Analog family calendar	Manage a family calendar together. Choose an analog family calendar if you feel analog is advantageous, e.g., because you can hang it at a prominent spot in your home. Both you and your partner should be involved in calendaring routines.
2.3.2 Digital family calendar	Manage a family calendar together. Choose a digital family calendar if you feel digital is advantageous, e.g., because you always have the calendar with you on your phone. Both you and your partner should have access to the digital calendar and be involved in calendaring routines.
2.3.3 Mobile communication	Use phone calls or messenger services to coordinate daily tasks, e.g., short term changes.
2.3.4 Cleaning schedule (written down)	Write down your weekly fixed routines (2.1.1), or write down a weekly fixed cleaning plan, or make a list of all tasks and who is responsible for which tasks.
2.3.5 Others	Use other coordination tools, such as to-do-lists or organization apps, if you like those.
**2.4 Reducing household workload**	
2.4.1 Reducing cleaning	Reduce time spent on cleaning. Leave some things undone. Lower your standards and relax if there is, e.g., no time to clean the windows this spring.
2.4.2 Reducing cooking	Reduce your time spent on cooking by, e.g., cooking fast meals, having bread for dinner, or ordering food.
2.4.3 Reducing grocery shopping trips	Reduce your time spent on grocery shopping by doing one big shopping trip per week instead of multiple small ones or ordering groceries.
2.4.4 Supporting household appliances	Use supporting household appliances, such as a thermomixer, dishwasher, or automatic vacuum cleaner.
2.4.5 Others	If you feel you can reduce time spent on household work by other measures, do so. E.g., if decorations are not important to you, do not put them up, so they do not have to be cleared away for cleaning.
**2.5 Distribution of (remaining) tasks**	
2.5.1 Designated responsibility	Distribute tasks by deciding which partner is solely responsible for which tasks. You can distribute the tasks according to preference or skill. Do not tell the other how or when to do their designated tasks. Leave the responsibility with the designated partner. This can be especially helpful to distribute routine housework. E.g., you are solely responsible for cleaning the kitchen and your partner is solely responsible for cleaning the bathroom.

2.5.2 Scheduled exchange (timely)	Switch tasks rhythmically, e.g., each second day. This can be especially helpful to distribute bringing/picking-up your child to/from daycare. E.g., you pick her/him up on Mondays, your partner picks her/him up on Tuesdays, and so on.
2.5.3 Situational exchange (spontaneously)	Switch tasks spontaneously if the situation requires it. This can be especially helpful in childcare if one partner is tired or stressed or if your child is sick.
2.5.4 Together (not distributed)	Do certain tasks together. This can be especially joyful in childcare, e.g., by playing together or doing the bedtime routine together. It can also be helpful for certain housework tasks, e.g., going grocery shopping.
**2.6 Execution of (remaining) tasks**	
2.6.1 Optimization of routes	Optimize your routes by, e.g., choosing a daycare facility close to home, buying things on the way home, or taking the bike instead of walking.
2.6.2 Rule of "30-seconds"	Do everything that can be done within 30 seconds immediately. E.g., if you've just drunk a glass of water, put it straight into the dishwasher instead of on top of it.
2.6.3 Utilizing mini-timeslots	Do tasks in short, unexpected windows of time, e.g., if you still have 10 minutes before you must leave your home.
2.6.4 Working in parallel in the household	Do tasks at the same time, e.g., one partner cleans the kitchen while the other cleans the bathroom during naptime of your child.
2.6.5 Moderate split-shift parenting	Bring your child to childcare (later) while your partner goes to work (earlier). In the afternoon your partner picks up your child (earlier) while you can stay at work (longer). This helps to fulfil work related requirements, reduce daycare time for the child, and increase parent-child time.
2.6.6 Combining tasks with leisure	Combine tasks with leisure, e.g., watch TV while ironing, listen to podcasts while cleaning, talk to your partner while folding laundry.
2.6.7 Others	If you can think of other strategies to be efficient in housework, implement those. E.g., find a designated place for important documents like the vaccination certificate. Always put them back to their designated place so that you can find them quickly when needed.

**2.7 Child's location during the execution of household tasks**	
2.7.1 Child sleeping	Do household tasks while your child is sleeping, e.g., in the mornings, evenings, or during naptime.
2.7.2 Childcare by other parent	Do household tasks while your partner is looking after your child.
2.7.3 Childcare by social network	Do household tasks while your child is with your social network, e.g., grandparents or friends.
2.7.4 Child at daycare	Do household tasks while your child is at daycare. This might be especially helpful for you if your paid work includes shift work.
2.7.5 Child involved in household chores	Involve your child in household tasks, e.g., take her/him with you when grocery shopping or encourage her/him to help with easy tasks.
**2.8 Assessment of the partner's childcare and housework abilities**	
2.8.1 Positive assessment	Assess your partner's childcare and housework abilities positively. This includes, e.g., to trust her/him to do things as well as you do them yourself, assess the way your partner completes a task favorably, and believe everything would go well if you weren't present yourself.
2.8.2 Positive assessment referring to childcare directly postpartum	Assess your partner's childcare and housework abilities positively directly postpartum. This includes sharing gender-typical tasks from an early stage. E.g., trust your partner to change the nappy, while you are responsible for breastfeeding.
**2.9 Regulating emerging imbalances in household and childcare**	
2.9.1 Regulating to prevent own overload	Recognize if you are taking over more tasks yourself and ask your partner to take over. E.g., formulate a specific wish in a certain situation or have a general conversation on the meta-level.
2.9.2 Regulating to prevent partner overload	Recognize if your partner is taking over more tasks and help her/him. E.g., take over a certain task, have a general conversation on the meta-level, or grant your partner a break.
2.9.3 Acceptance of temporary fluctuations	Accept temporary fluctuations if the distribution of tasks is balanced on average. Do not exploit your partner's trust that fluctuations will ultimately even out.

Combine strategies, even if they feel opposing. E.g., combine foresighted planning with flexibility, regular with situational exchange of tasks, and preventing own with preventing partner overload. Try to find a balance between the seemingly opposing poles. For more details, see main article.
